# The Effects of Silver Nanoparticles Compositions on the Mechanical, Physiochemical, Antibacterial, and Morphology Properties of Sugar Palm Starch Biocomposites for Antibacterial Coating

**DOI:** 10.3390/polym12112605

**Published:** 2020-11-06

**Authors:** A. Rozilah, C. N. Aiza Jaafar, S. M. Sapuan, I. Zainol, R. A. Ilyas

**Affiliations:** 1 Laboratory of Biocomposite Technology, Institute of Tropical Forestry and Forest Products (INTROP) Universiti Putra Malaysia, UPM Serdang, Selangor 43400, Malaysia; rozilahabdullahsilver85@gmail.com (A.R.); sapuan@upm.edu.my (S.M.S.); ahmadilyasrushdan@yahoo.com (R.A.I.); 2 Department of Mechanical and Manufacturing Engineering, Universiti Putra Malaysia, UPM Serdang, Selangor 43400, Malaysia; 3 Advanced Engineering Materials and Composites Research Centre (AEMC), Department of Mechanical and Manufacturing Engineering, Universiti Putra Malaysia, UPM Serdang, Selangor 43400, Malaysia; 4 Faculty of Science and Mathematics, Sultan Azlan Shah Campus, Universiti Pendidikan Sultan Idris, Proton City, Tanjung Malim 35900, Malaysia; ismail.zainol@fsmt.upsi.edu.my

**Keywords:** silver nanoparticles, sugar palm starch, sugar palm nanocrystalline cellulose, microorganism, antibacterial film

## Abstract

Antibacterial sugar palm starch biopolymer composite films were developed and derived from renewable sources and inorganic silver nanoparticles (AgNPs) as main ingredients for antibacterial coatings. The composite films were produced by solution casting method and the mechanical and physicochemical properties were determined by tensile test, Fourier Transform Infrared (FTIR) analysis, thermal gravimetric analysis (TGA), antibacterial screening test and field emission scanning electron microscopy (FESEM) images. It was found that mechanical and antibacterial properties of biocomposite films were improved after the addition of AgNPs compared with the film without active metals. The weakness of neat biocomposite films was improved by incorporating inorganic AgNPs as a nanofiller in the films’ matrix to avoid bacterial growth. The results showed that the tensile strength ranged between 8 kPa and 408 kPa and the elasticity modulus was between 5.72 kPa and 9.86 kPa. The addition of AgNPs in FTIR analysis decreased the transmittance value, caused small changes in the chemical structure, caused small differences in the intensity peaks, and produced longer wavelengths. These active films increased the degradation weight and decomposition temperature due to the more heat-stable AgNPs. Meanwhile, the average inhibited areas measured were between 7.66 and 7.83 mm (*Escherichia coli*), 7.5 and 8.0 mm (*Salmonella cholerasuis*), and 0.1 and 0.5 mm for *Staphylococcus aureus*. From the microscopic analysis, it was observed that the average size of all microbes for 1 wt% and 4 wt% AgNPs ranged from 0.57 to 2.90 mm. Overall, 3 wt% AgNP nanofiller was found to be the best composition that fulfilled all the mechanical properties and had better antimicrobial properties. Thus, the development of an organic-inorganic hybrid of antibacterial biopolymer composite films is suitable for antibacterial coatings.

## 1. Introduction

The microbial contamination of food is one of the main problems in the food industry; considering the waste of spoiled products and the implications for public health, the production of active packaging would be beneficial for both issues [1,2,3,4,5]. Nowadays, environmentally friendly food packaging with good antimicrobial and barrier properties is seen to be the solution to reduce environmental problems, extend shelf life, and improve the food storage environment [6]. Organic-inorganic hybrid material is normally used in the production of multiple packaging plastic applications for improvements in their properties. The applications of silver nanoparticles (AgNPs) in antibacterial coating have been widely used to avoid the build-up of pathogenic bacteria and spoilage fungi. It has been suggested that AgNPs possess antibacterial properties against various microorganisms, such as Gram-negative *E. coli* and Gram-positive *S. aureus*, and that smaller size AgNPs exhibit more efficient antibacterial activity due to their high surface area and better contact with microbes [7].

Antibacterial coating acts to reduce the inhibition or retard the growth of microorganisms that might be present in packed food or on the surface of the packaging material itself. Active packaging with antibacterial properties has been developed in food packaging, especially with the addition of nanosilver, which might increase the quality of the product and its shelf life, and prevent spoilage caused by bacterial action. The tensile strength and molecular orientations in film metrics were improved using AgNPs agents to reduce the food’s moisture content. Furthermore, a good adhesion between the filler and the matrix leads to a resistant interface [8], which consequently reinforces the matrix and enhancing its properties. Inorganic AgNPs were used because of their high surface to volume ratio with increased surface reactivity, and they are more heat stable at high temperatures compared with organic antimicrobials. By introducing a metal agent, this will create a strong reinforcement bonding which possess the ability to reduce the water content as well as providing protection from bacterial attack. Silver is reasonably effective at penetrating biofilms, which is a drawback to many molecular antimicrobials [9]. In studies, AgNPs have been used together with biopolymer composites materials because they are cost-effective; stable; and can be easily incorporated into numerous materials, such as textiles and plastics. Among them, silver nanoparticles are one of the most widely used antimicrobial substances in antibacterial coating applications [10].

Starch is composed of both linear and branched polysaccharides, and is known as amylose (15–30%) and amylopectin (70–85%), respectively [11,12,13,14,15]. Starch can be extracted from sugar palm trunk (*Arenga Pinnata*), a multipurpose material with several industrial uses, especially in plastic packaging. Sugar palm starch has been extensively used to produce bio-based starch films, and the results show that these carbohydrates are promising materials in this regard [12,13,16,17,18,19,20,21,22,23,24]. The films developed from sugar palm starch are described as non-toxic, colorless, biodegradable, tasteless, odorless, and isotropic. In a previous study conducted by Ilyas et al. [22], sugar palm starch films plasticized with glycerol and sorbitol and reinforced with sugar palm nanocellulose were developed to enhance their mechanical and barrier properties [25,26,27,28]. The mechanical properties of starch biopolymers can be improved with the addition of natural fibres [11,29]. The results reported were important for the continuity of the research because they gave information about the optimal formulation to produce composite films with better mechanical, thermal, and water barrier properties. Therefore, currently the authors are trying to incorporate antimicrobial agents into the formulation of sugar palm starch films, since carrying natural additives could be considered a new tendency of functional antibacterial coatings in the near future. Active packaging offers microbial safety for consumers by reducing, retarding, or inhibiting the growth of microorganisms, and then could extend the shelf life of the packaged food [30,31,32,33].

The starch trunk was covered with long black fibres known as sugar palm fibre (SPF) [13,34,35]. SPF was extracted using delignification, mercerization, and acid hydrolysis processes to form sugar palm nanoparticles crystalline cellulose (SPNCCs) [22,25,28]. SPNCCs possess the advantages of being renewable, biodegradable, inexpensive, and abundantly available, thus they have a promising future in the field of the biocomposite industry [25,36]. Researchers have shown improvement in the water vapor and gas barrier properties by developing nanocellulose composite films [37,38,39]. Moreover, cellulose fibres have specified properties, such as high specific stiffness, low density, and biodegradability [34,40,41,42,43]. The SPNCCs function as fillers on the core side that control the absorption of moisture and strengthen the polymer metric [44]. The addition of nanofibers enhanced the strength of the molecular arrangement orientations in the films, and thus increased the films’ strength and elasticity. It was considered that the mechanical properties of biocomposite films depend on the loading and orientation of fibres in biopolymer [29,45,46,47,48,49,50].

The effectiveness of AgNPs as antibacterial agents in different compositions was reported against *Escherichia coli*, *Staphylococcus aureus*, and *Salmonella cholerasuis* microbes [51]. Moreover, studies on AgNPs’ antibacterial activity against Gram-positive *S. aureus* and Gram-negative *E. coli* showed that the inhibition of growth in case of *S. aureus* was less remarkable, while *E. coli* was inhibited at low AgNP concentrations [51]. All the microbes were composed of a peptidoglycan layer with different thicknesses, and silver nanoparticles were able to rupture the bacterial wall. It was reported that the Gram-negative bacteria were thinner and easier to damage compared to those that were Gram-positive with thicker cells walls [10]. Gram-negative bacteria (e.g., *E. coli*) are generally more susceptible to silver treatment than Gram-positive bacteria (e.g., *S. aureus*) because the transport of positively charged silver ions across the thicker, peptidoglycan-rich outer membranes of Gram-positive bacteria is slower relative to transport across the thinner membranes of Gram-negative specimens [10]. Highly reactive metal oxide nanoparticles exhibit excellent antimicrobial effects action against Gram-positive and Gram-negative bacteria [52]. Additionally, the antimicrobial effects might reach maximum levels by the direct incorporation with the biopolymer material in the film matrix.

The incorporation of bioactive agents, including antimicrobials, into polymers has been commercially applied in pesticide delivery, household goods, textiles, biomedical devices, and a few food-related applications [53,54]. These antibacterial films with AgNPs are homogeneous and stable for a longer duration before they biodegrade. Due to their wide range of uses in both domestic and industrial applications, it is important to consider the possible releases of silver nanoparticles (AgNPs) into the environment and the possible toxicity of AgNPs to humans, aquatic organisms and the environment. Research was used to introduce and investigate the effects of AgNPs as active ingredients by combining them with biodegradable polymer starch and nanocellulose fibre in packaging film. Work has also been carried out to reveal the effects of silver nanoparticle (AgNP) compositions on the physicochemical, mechanical, and antibacterial properties of active films. The changes in the nanoparticle sizes and the microstructures of dry bacterial colonies were investigated to view their effects on AgNPs compositions.

The widely used synthetic polymers in the antibacterial coating system can be reduced and replaced by biodegradable sources in order to maintain environmental sustainability [55,56]. The combination of biopolymer composites with silver nanoparticles (AgNPs) for antibacterial effects is to prevent unwanted bacterial formation, thus it will reduce the toxicity effects and prevent wastage. Based on a literature survey, none of the previous reports have dealt with the effects of AgNPs on the mechanical, physiochemical, antibacterial, and morphology properties of sugar palm nanocellulose (SPN)-reinforced sugar palm starch (SPS) biopolymer composites. Thus, this study focused on investigating the potential effects of using AgNPs as fillers on the thermal stability, flammability, and morphological properties of SPN/SPS biopolymer composites at various loadings.

## 2. Methodology

### 2.1. Materials

Sugar palm starch (SPS) and sugar palm nanoparticles crystalline cellulose (SPNCCs) were prepared at the Laboratory of Biocomposite Technology, Institute of Tropical Forestry and Forest Products (Introp, UPM, Serdang, Malaysia). Sorbitol and glycerol were purchased from R&M Chemicals Sdn. Bhd. (Selangor, Malaysia). Inorganic silver nanoparticles (AgNPs) with sizes in the range of 20–40 nm was purchased from Alfa Aesar (Selangor, Malaysia). The microbes *Escherichia coli* (ATCC 25922), *Staphylococcus aureus* (ATCC 43300), and *Salmonella cholerasuis* (ATCC 10708) were supplied and prepared in fresh conditions at the Microbial Culture Collection Unit (UNiCC) at the Institute of Bioscience, Universiti Putra Malaysia.

### 2.2. Sugar Palm Starch Extraction and Preparation

Sugar palm starch (SPS) was extracted from the stem of a matured sugar palm tree. Workers cut the tree using a chainsaw so that the mixture of woody fibre and starch powder could be collected from the interior part of the stem. The washing process was carried out by adding water to the mixture and then kneading it by hand or using a specially designed machine to extract the starch from the mixture. The mixture was then filtered using a sieve, where the fibre remained at the top of the sieve and starch granules flowed with the water into a container. The starch was separated from the water, where water was poured slowly until it reached the level of the starch, as it is denser than water. Fibre residues that are by-products were isolated from wet starch. Then, the wet starch was let to sun-dry for 30 min and was finally dried in oven at 120 °C for 24 h [57].

### 2.3. Sugar Palm Fibre (SPF) Extraction and Preparation

SPF is located on the trunk of the sugar palm tree as a natural woven shape fibre. SPF wraps up the tree trunk and a worker used an axe to cut and remove it from the tree. Then, the SPF was ground and filtered to a 2 mm size.

### 2.4. Cellulose Extraction

The two main processes that were carried out to extract the cellulose fibres from the sugar palm fibres (SPF) were delignification and mercerization. Lignin was removed from SPF to get holocellulose through chlorination and bleaching processes in accordance with ASTM D1104-56 (1978). According to ASTM D1103-60 (1977), holocelluloses should undergo further treatment to produce α-cellulose [58].

### 2.5. Isolation of Sugar Palm Nanocrystalline Cellulose (SPNCCs)

SPNCCs was prepared by acid hydrolysis. Mechanical stirring was set up with a rotation speed of 1200 rpm at a 45 °C temperature for a 45 min time period to stir the aqueous H_2_SO_4_ (60 wt%) mix with cellulose. The ratio of cellulose to H_2_SO_4_ solution was 5:100 (wt%). Then, a washing process took place for the hydrolyzed cellulose 4 times by centrifugation (6000 rpm, 20 min, and 20 °C) to ensure that all the leftover H_2_SO_4_ was removed. The cellulose was then dialyzed by distilled water until it reached a neutral pH (6.5 to 7). Then, it was sonicated for 30 min by using a sonicator. Finally, the cellulose was freeze-dried and stored in cool place prior to analysis and application as a reinforcement in starch film. The isolated SPNCCs were found to have lengths and diameters of 130 ± 30 nm and 9 ± 1.96 nm, respectively [25].

### 2.6. Preparation of Antibacterial Biopolymer Composite Films

The solution casting method was used in the fabrication of antibacterial biopolymer composite films. For the first step, 1.5 g of glycerol, 0.05 g of SPNCCs, and AgNPs were mixed with different compositions (0–4 wt% on the starch basis) in 190 mL of distilled water. The solutions were stirred for 5 min and ultrasonificated for about 15 min. The solutions were heated in a water bath at 85 °C, added to 10 g of SPS and 1.5 g of sorbitol, and then continuously stirred for 30 min. The solution was transferred into a petri dish and dried in a convection oven at a temperature of 45 °C for about 24 h. After cooling, the films were peeled off and kept in desiccators for 48 h to control the moisture content. As shown in Table 1, the material compositions were fixed in glycerol, sorbitol, SPS, and SPNCCs and only varied in the weight percentage of AgNPs. The weight percentages of AgNPs were derived from the total amount of mixing material added. The weights of SPS and SPNCCs in these formulations were fixed for the same effects and changed only for the AgNPs weight compositions. The reasons for this were to maintain the same composition of biopolymer composites in all samples and to observe the effects of the AgNPs at different weight percentages. The best weight composition for an antibacterial coating with less elasticity, a high strengthm and good antibacterial protections can be observed by varying the weight percentage of AgNPs.

### 2.7. Mechanical Properties

Tensile properties such as the tensile strength, tensile modulus, and elongation at break of the films were tested according to the standard method of D882-02 (ASTM, 2002) using the Instron 3365 universal testing machine (High Wycombe, UK) with a load cell of 5 kN. The rectangular strips films with dimensions of 10 mm × 70 mm were clamped between two tensile grips (model 2710-105) with an initial gauge length of 30 mm and pulled at a crosshead speed of 2 mm/min. The force (N) and deformation (mm) of the samples were automatically recorded and measurements were carried out in 10 replicates for each composition.

### 2.8. FTIR Analysis

The identifications of functional groups in the antibacterial biopolymer composite films were detected by Fourier Transform Infrared (FT-IR) spectrometer (Nicolet iS10, Thermo Scientific, Waltham, MA, USA). FTIR spectra were obtained at wavenumbers ranging from 4000 to 500 cm^−1^, with 16 scans recorded at a 4 cm^−1^ resolution.

### 2.9. Thermal Properties

The weight changes and decomposition temperatures were detected by thermogravimetric analysis (TGA), (TGA-IR-Nicolet iZ10, Thermo Scientific, Waltham, MA, USA). About 10 mg or less of film samples were heated from room temperature until a 600 °C temperature with a heating rate of 10 °C/min under a cooling nitrogen flow. The weight loss (%) was determined from the TGA curve and the maximum decomposition temperature was calculated from a TGA derivative (DTG curves).

### 2.10. Antibacterial Properties of Composite Films

Antibacterial biopolymer composite films were tested using antimicrobacterial screening tests with fresh spread bacterial cultured in wet agar to measure the size of the inhibited area. Antibacterial biopolymer composites with thicknesses of 0.180 to 0.250 ± 0.05 mm were cut into circular discs and immersed on a cultured plate of 0.5 mL McFarland agar broths containing three types of bacteria, which were *Escherichia coli,* ATCC 25922 (Gram-negative), *Staphylococcus aurous,* ATCC 43300 (Gram-positive) and *Salmonella cholerasuis,* ATCC 10708 (Gram-positive), with 1 × 10^8^ CFU/mL standard concentrations using 4 replicates for each types. The plates were inverted and incubated with temperature around 37 °C for 18 h. The results were collected after 24 h of the incubation process.

### 2.11. Microstructure Study of AgNPs and Bacterial Colonies

The microstructure of AgNPs nanoparticles and film surface containing bacterial colonies were analyzed using Field Emission Scanning Electron Microscopy (FESEM) from the JEOL model JSM-7600F (Akishima, Tokyo, Japan) equipped with an electron gun emission field with the accelerating voltage of 5.0 kV. Samples were placed on the scanning plates by using black conductive tapes to avoid moving. All the samples were then coated with gold to prevent over-charging and sample damage during the scanning processes. The nanostructure was measured in terms of the nanoparticles size on the surfaces and matrices of antibacterial films. The bacterial colonies and length and diameter of dry microbes were analyzed and measured to evaluate the size differences.

### 2.12. Statistical Analysis

Statistical analysis was performed using the statistical analysis ANOVA-one way repeated measurement in OriginPro 9.0 version (OriginLab Corporation, Northampton, MA, USA). The significant differences between the mean values were further determined by the multiple-range test at a 95% confidence level (*p* < 0.05). The values were presented as the mean ± SD (standard deviation).

## 3. Results and Discussion

### 3.1. Antibacterial Properties

The effectiveness of the antibacterial activity of the films was dependent on the AgNP composition and the size for the capability of strong inhibition effects [59]. An individual AgNP nanometal can attack a bacterial membrane with scale size of 100 nm and below for more effective results. More precisely, silver nanoparticles with sizes between 10 and 15 nm have increased stability and biocompatibility and enhanced antimicrobial activity [60]. The smaller the particles size of AgNPs, the easier silver ions penetrate the cells wall, but, in fact, increasing the composition will creates more agglomeration and increases in particle size. Generally, the antibacterial activities of AgNPs in films are affected by the availability of ionic silver for bacterial contact [61], the composition of AgNPs added, and agglomerations.

The AgNPs will react with cultured bacterial on the wet agar medium by killing all the pathogens around the samples, resulted in increasing the inhibited area measured. The silver ions accumulated in the bacterial cytoplasmic membrane, causing a significant increase in membrane permeability and leading to cell death. The antimicrobial mechanism of AgNPs has also been suggested to be related to membrane damage due to free radicals derived from the surface of the nanoparticles [51]. It has been proposed that positively charged silver ions can interact with negatively charged bio macromolecular components (phosphate and disulfide or sulfhydryl groups of enzymes) and nucleic acids, causing structural changes and deformation in bacterial cell walls and membranes that lead to the disruption of metabolic processes, followed by cell death [60,62].

**Figure 1:**
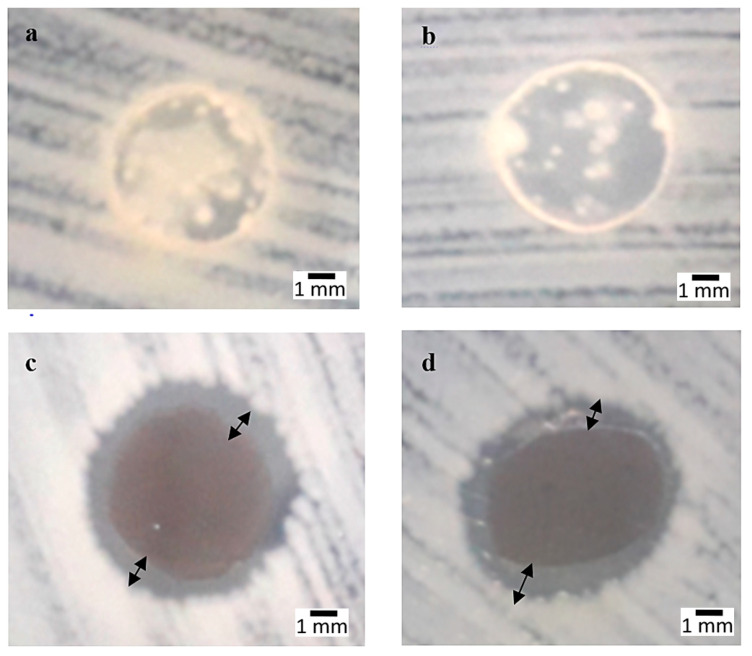
*Escherichia coli* strain: (**a**) Sugar palm starch (SPS), (**b**) 0 wt%, (**c**) 3 wt%, (**d**) 4 wt% compositions.

Figure 1 shows the inhibited surfaces of antibacterial biopolymer composite films, displaying a higher activity at 3 wt% and 4 wt% compositions in *E. coli*. For the compositions of 3 wt%, the average inhibition area was 7.83 ± 0.166 mm, and for 4 wt% it was 7.66 ± 0.166 mm. Moreover, the results indicated that no inhibition area appeared on SPS for 0 wt%, but a slight change to the yellowish color around the circular disc was observed. This shows that SPS and SPNCCs in antibacterial films also contributed in the deactivation of bacterial growth on samples, but with lesser effects. A little inhibited zone appeared with an unmeasured diameter on 1 and 2 wt%, but the inhibition areas were larger, as observed in the higher compositions of 3 and 4 wt% due to the smaller sizes and strong activation of silver ions. In the higher composition of AgNPs at 3 wt% and 4 wt%, the media were packed and dense, hence requiring the AgNPs to produce stronger silver ions to be able to penetrate the cell walls of positive and negative types of bacteria, which then initiated the large value of the inhibition area. The differences on the inhibition area might have been caused by AgNPs that acted as a discontinuous barrier for the diffusion of the water vapor in the film matrix, thus increasing the path length of the diffusion [63].

**Figure 2:**
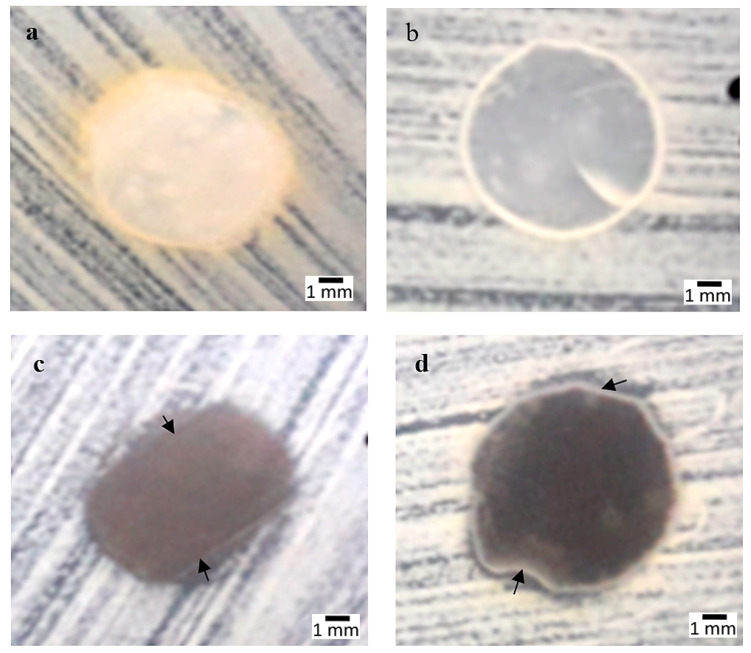
*Staphylococcus aureus* strain: (**a**) Sugar palm starch (SPS), (**b**) 0 wt%, (**c**) 3 wt%, (**d**) 4 wt% compositions.

From Figure 2, it can be observed that high compositions of AgNPs gave weaker effects towards *Staphylococcus aureus* bacteria due its thicker cell walls. Gram-positive bacteria were less permeable to silver ions compared to Gram-negative due to the same reason. The differences in the antimicrobial activity of AgNPs between Gram-positive and Gram-negative bacteria could be partly explained by the difference in the structure of their cell walls. The walls thickness can be associated with the different structure of the outer membrane and peptidoglycan layer, thus Gram-negative bacteria (*E. coli* and *Salmonella*) were more sensitive to AgNPs than the Gram-positive ones (*S. aureus*) [51,59,64].

The measurement of the inhibited area measured in 3 wt% AgNPs in *S. aureus* was 0.1 ± 0.00 mm and for 4 wt% it was 0.5 ± 0.00 mm. The SPS and 0 wt% samples showed yellow precipitation on the surface, which might be caused from the building up of bacterial colonies due to the fewer silver ions released. The walls of *Staphylococcus aureus* (+ve) were thicker compared to those of *Escherichia coli* (−ve) and *Salmonella cholerasuis* (−ve), thus the penetration of silver ion was less compared to negative types of microbes. Gram-positive bacteria are composed of a thick peptidoglycan (20–80 nm) layer consisting of linear polysaccharide chains cross linked by short peptides, forming a complex structure that makes it more difficult for silver ions to penetrate into Gram-positive bacteria [65].

**Figure 3:**
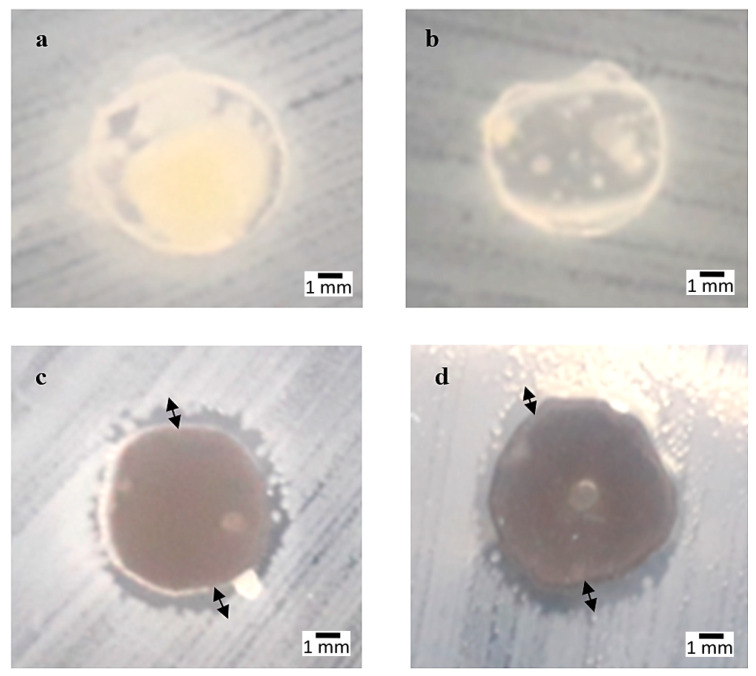
*Salmonella cholerasuis* strain: (**a**) Sugar palm starch (SPS), (**b**) 0 wt%, (**c**) 3 wt%, (**d**) 4 wt% compositions.

Referring to Figure 3, *Salmonella cholerasuis* produced a large area of inhibition with a similar percentage value to *E. coli* at a higher percentage. SPS and 0 wt% were observed to appear in a yellowish color and were similar for all types of microbes tested; meanwhile, 3 and 4 wt% produced a larger inhibited area around the circle. The average area of inhibition measured in 3 wt% tested with *Salmonella* was within 7.5 ± 0.288 mm and in 4 wt% this was increased to 8.0 ± 0.288 mm. Te average inhibition area for 4 wt% of *E. coli* and *Salmonella* were slightly higher compared to 3 wt% and not many differences were observed, as they were derived from the same negative types of microbes.

Increasing the weight composition of AgNPs would produce an effective result, but the solution became dense and more agglomerated, causing an increase in the size of nanoparticles and level of toxicity. Moreover, the size and shape of the AgNPs played an important role in the enhancement of antimicrobial activity [66]. It can be concluded that, higher compositions gave smaller nanoparticles size and more effective in deactivation of active pathogen for reducing bacterial attachments. Overall results showed 3 wt% of AgNPs was the best composition and recommended to be used for antibacterial film. This was due to higher measurements of inhibition area with smaller nanoparticles size that appeared to have the same effectiveness as 4 wt% with less toxicity effects.

### 3.2. Thickness of Antibacterial Films

The thickness of antibacterial biopolymer composite films was increased by increasing AgNPs compositions from 0 wt% to 2 wt%, ranging from 2.013 × 10^−1^ ± 9.2 × 10^−3^ mm to 2.205 × 10^−1^ ± 9.2 × 10^−3^ mm. Meanwhile, the thickness for 3 wt% decreased by 1.674 × 10^−1^ ± 9.2 × 10^−3^ mm due to the material mixing that had reached the maximum compositions level and the effects of heat stable AgNPs resulted in reducing the thickness. Meanwhile, 4 wt% showed a little increasing in thickness value by 1.811 × 10^−1^ ± 9.2 × 10^−3^ mm due to the stability of AgNPs by increasing the weight volume.

Increasing the compositions of AgNPs will reduce the thickness of the antibacterial biopolymer composite films affected by the heat stability of nanometals. The biopolymer composites are now more compatible and easier to biodegrade in the environment. This condition happened at high AgNP compositions because the nanometals on the films’ matrix absorbed heat and these compositions reached the maximum mixture value. The 3 wt% was the lowest in terms of thickness for antibacterial films and achieved the recommended weight percentage for the production of antibacterial films.

### 3.3. FTIR Analysis

FTIR analysis was used to identify the functional groups that appeared in the composite films. The common functional group detected were carbonyl (C=O), hydroxyl (O–H), carbon double bond (C=C) and carboxylate group. The antibacterial biopolymer composite films showed peaks within range of 3495–524 cm^−1^ caused by organic functional group that appeared in SPS and SPNCCs in different wave number. Incorporation of AgNPs composition into biopolymer composite films led to decreased in the peak intensity and area under the peak, which could be attributed to the strong interaction between the carboxylate group, O–H groups and Ag^+^ group. Pandey et al. [67] found that the O–H group of polymer had efficient coordination ability with silver ions.

**Figure 4:**
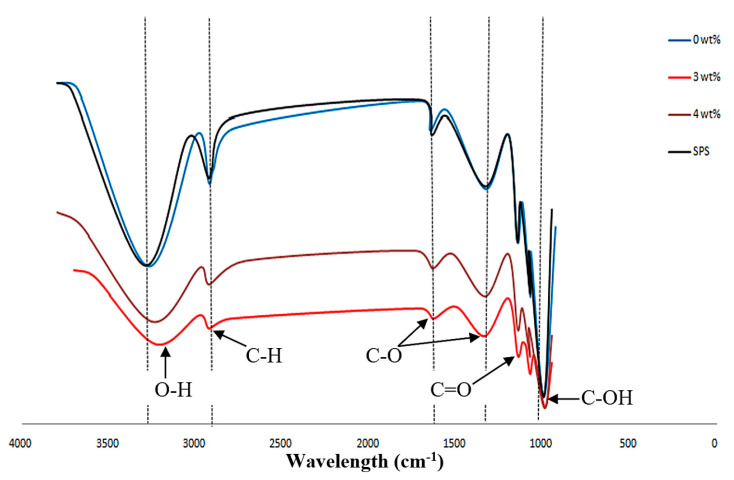
FTIR spectrum of the nanocomposite films with different AgNPs compositions.

Figure 4 shows the common functional group in polysaccharide and their location in different wavelength. The antibacterial biopolymer composite films showed peaks of 3332–3235 cm^−1^ which indicated the stretching mode of OH group in organic biopolymer composites. Similar report was studied by Jordana et al. [68], the intense and broad adsorption peak between 3100 and 3500 cm^−1^ were associated with the stretching vibrations of the hydroxyl group. Meanwhile, the absorptions peak within 2927–2898 cm^−1^ contributed to the stretching of the C–H bonding. The adsorptions at 2850–2930 cm^−1^ were due to the stretching vibrations of the C–H groups present in the biopolymer structure [69].

The peaks of 1654–1075 cm^−1^ were derived from the C–O stretching groups in the nanocellulose of SPNCCs. The appearance of nanocellulose at peak 1634.9 cm^−1^ corresponded to the C–O stretching [70]. The observed peaks at 1338–896 cm^−1^ were derived from the carbonyl (C=O) stretching region in the molecular structure. The C=O carbonyl stretching bands from the glucose of the cellulose appeared at 1645 cm^−1^, and a C–O–C stretching region at 1300–900 cm^−1^ was also observed [71]. Lastly, the region between the peaks of 1030 to 758.05 cm^−1^ indicated the presence of C–OH bending. In addition, the band at 1018 cm^−1^ represented the bending vibrations of the C–OH bond [72].

The results indicated that he addition of AgNPs at higher volumes decreased the transmittance value, caused small changes in the peak intensities, and caused the appearance of AgO precipitations. It was observed that the 0 wt% and SPS samples showed higher transmittances and wavelengths due to the large amount of polysaccharide molecules; meanwhile, the 3 wt% and 4 wt% showed a reduction in the peak transmittance and shorter wavelengths due to the dense and high concentration of silver ion. For 3 wt% AgNPs, the volume ratio reached the maximum and showed a balance coordination of molecular arrangements with the lowest transmittance value, thus making it a suitable composition for antibacterial films.

### 3.4. TGA Analysis

Thermogravimetric analysis (TGA) was used to study the effect thermal stability of AgNPs’ composition in degradation weight during heating processes. The TGA curve presented three stages: (i) the loss of absorbed water; (ii) structural water, SPS, SPNCCs, and AgNPs; (iii) the decomposition of SPS and SPNCCs molecules. This first step of the degradation was mainly due to the moisture loss from the films [63]. The second degradation mainly involved the dehydration reaction and the formation of volatile matter [73]. The temperature at which maximum degradation occurred at approximately 300 °C was due to the decomposition and depolymerization of starch carbon chains [74,75].

**Figure 5:**
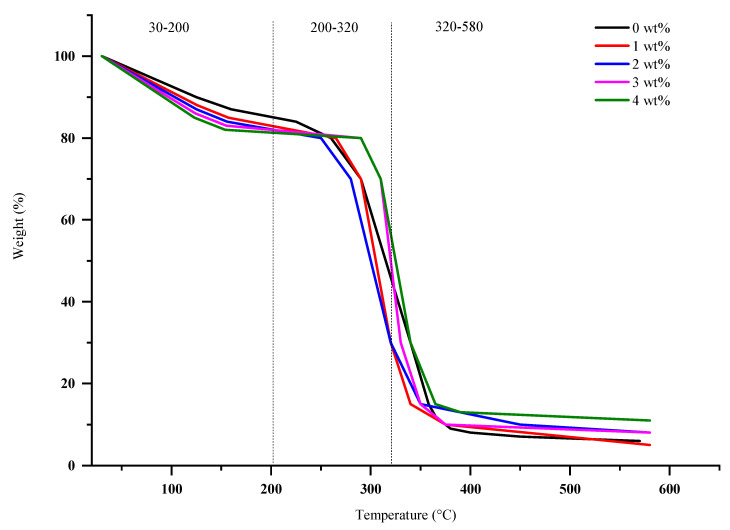
Degradation phase differences in the TGA analysis.

Figure 5 shows the three main stages of weight degradations in the TGA analysis. Referring to Figure 5, the first stage for 0–2 wt% in weight loss occurred between 30 and 165 °C, associated with water evaporation in composite films, which accounted for about 18.35%, 16.96%, and 18.46% of the weight loss. Meanwhile, at 3 wt% and 4 wt%, the values were counted to increase by 19.64% and 20.67% weight loss, respectively, which occurred in the temperature region between 30 and 200 °C. The results showed that the degradation weight and temperature were increasing for higher compositions due to the nanosilver’s ability to store heat at a high volume. All the samples showed an initial weight loss in the region of 39 to 130 °C, which can be ascribed to the evaporation loss of the physically weak and loosely bound moisture on the surfaces of the composite films [76].

The combined of second stage and third stage weight loss observed from 0 to 2 wt% occurred at region 165−315 °C and 315−580 °C contributed to 75.32%, 77.63% and 74.20% of weight loss. Otherwise, the region between 200−320 °C and 320−580 °C that appeared for 3 wt% and 4 wt% was observed to decrease in value by 69.87% and 67.49% weight loss, respectively. The decrease in the degradation value were caused by the higher composition of AgNPs, more heat storage, and reduced melting of biopolymer composites. The degradation that occurred at approximately 200–270 °C might also be associated with decomposition of hemicellulose and the early stage of cellulose decomposition [77]. Above 480 °C, the degradation involved the decomposition of carbonaceous matter [78].

The results indicated that the addition of AgNPs to the biopolymer composites film increased the thermal stability as shown in Figure 5, which were mainly due to the heat stability of metallic silver. The addition of AgNPs in the compositions caused the thermal stability to increase due to the greater heat stability of metallic silver [63]. The weight degradation of antibacterial films was decreased prior to the heat stability of AgNPs by increasing the compositions. It was found that 3 and 4 wt% were the best compositions for antibacterial films due to their stability at high temperatures and reduced percentage of weight loss during processing. The recommended composition was 3 wt% due to it having less heat storage compared to 4 wt%, but their degradation weights were almost similar.

### 3.5. Mechanical Properties of Antibacterial Films

The mechanical properties of the films were studied to determine the values of important parameters such as tensile strength, modulus of elasticity, strain and percent of elongation. Tensile strength and extension are important film parameters which can be used as indicators for the film to maintain the film integrity and to withstand environmental stress during antibacterial coating applications [79].

**Figure 6:**
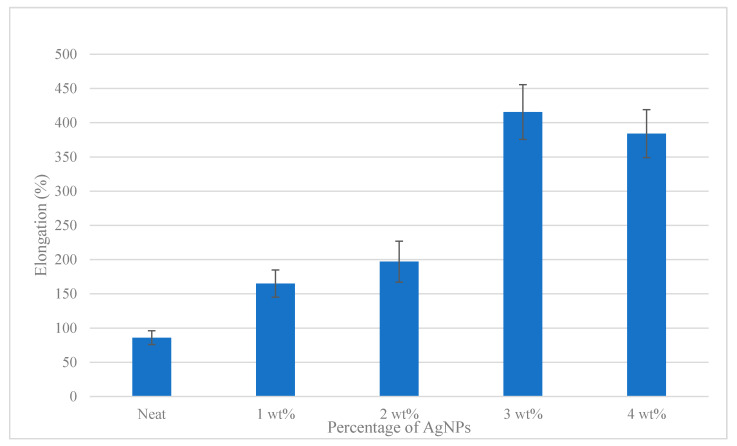
The effects of AgNP fillers on the elongation measurements.

Figure 6 shows that the elongation measurements were higher at a high composition of AgNPs. As observed in Figure 6, 3 wt% showed the highest elongation percentage and shortest time to rapture with value 415%. Other composition such as 0 wt%, 1 wt% and 2 wt% showed increasing value within 86%, 165% and 197%, respectively. It can be observed that the elasticity values of the films were increased when the percentage of the AgNPs was increased. Moreover, 4 wt% showed a decreasing in value by 38.84%. The excess of AgNP loading reduced the molecular mobility of the SPS matrix, making the nanocomposite materials stiffer. Therefore, the AgNPs/SPS nanocomposite films became more resistant to break, stiffer, and less stretchable compared to the control SPS films. The mechanical properties of the films were closely related to the distribution and density of the intra- and intermolecular interactions between the polymer chains in the film matrix [80]. Hence, it can be concluded that the elongation at break was reduced because of the rigid structure of the nanofiller and this was supported with past outcomes published in the literature by Sondi et al. [32], Savadekar et al. [81], and Jo et al. [82].

**Figure 7:**
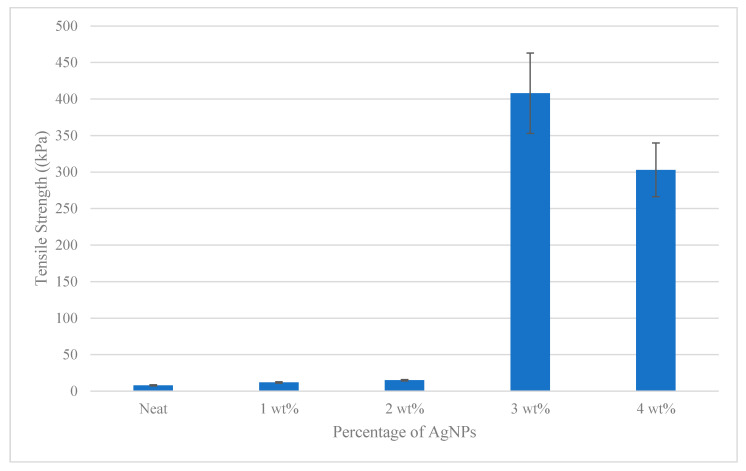
The strength of antibacterial films containing AgNPs nanometals.

Figure 7 shows that AgNPs affected the tensile strength of antibacterial films to be more ductile at high compositions. Referring to Figure 7, tensile strength in 0 wt% until 4 wt% were increasing by weight percentage within 8 kPa to 408 kPa. The addition of 1 wt% AgNPs within the films increased the tensile properties of the film by 165 kPa. As described, the morphological observation and optical property test results showed that the AgNPs were nanoscale in size, had large aspect ratios, and were distributed homogeneously within starch. Thus, the strong interaction between AgNPs and SPS particle formed during the film-making process resulted to an obvious improvement in the mechanical properties of the resulting nanocomposite films. This observation of the behavioral increment in tensile and modulus can be attributed to the favorable interaction between the AgNPs and SPS polymer matrices, which facilitated adequate interfacial adhesion. The reinforcement of AgNPs and SPS showed a similar effect with the previously reported result of AgNP-reinforced corn starch [83], sago starch [84], and rice starch [64] on the mechanical properties of starch-based nanocomposites.

Nevertheless, the tensile strength of the 4 wt% film (303 kPa) was lower compared to that of the 3 wt% film. This phenomenon might be due to the agglomeration and uneven distribution of the AgNPs within the SPS starch, in which the AgNPs failed to act as a reinforcement agent in starch biopolymer. Besides, it might also due to the excess AgNPs content that was likely causing large agglomerate formation, phase separation, and poor particle distribution, which led to poor mechanical properties. The strength of antibacterial films was reduced as increasing the AgNPs compositions, thus 3 wt% was the best composition to create an antibacterial film.

**Figure 8:**
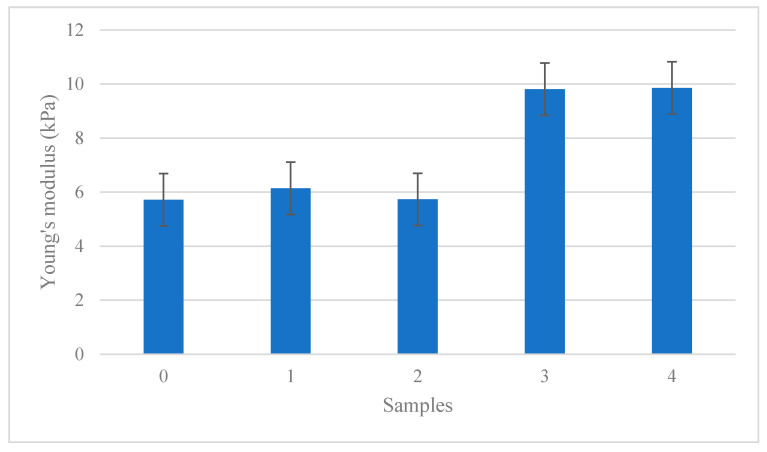
The elasticity effects of the antibacterial films by composition.

Figure 8 shows the modulus of elasticity or the Young’s modulus effects of antibacterial films that were increased in values, showing that the increase in AgNPs created less elastic films. Referring to Figure 8, the elasticity of antibacterial films from 0 wt% until 4 wt% showed increasing values from 5.72 to 9.86 kPa. Meanwhile, antibacterial films without AgNPs at 0 wt% showed a lower value of 5.72 kPa compared with that of 1 wt%, at 6.14 kPa. This caused additional AgNPs in 1 wt% to create more elastic properties compared to the film without the addition of AgNPs. Furthermore, the values for 2, 3, and 4 wt% were 5.73, 9.81, and 9.86 kPa, respectively. These results indicated that the AgNPs composition influenced the modulus of antibacterial films to form less elastic material.

Increasing the value of the tensile strength, elongation, and elasticity resulted in a reduction in the mechanical properties of antibacterial biopolymer composite films. This indicated that the elasticity influenced the average value observed for the tensile strength and elongations. The orientation of agglomeration, room temperature, and storage period might contribute to the creation of hillocking and nanoparticle/nanofiber growth. Besides that, the nanoparticle boundary strengthening contributed to the weakening of material bonding, thus reducing the ability to deform consistently. The overall histogram values show that 3 wt% and 4 wt% were the highest values, at which the antibacterial films resulted in being easy to break, less plasticized, more ductile, and less elastic with a smooth surface of films. The value of 3 wt% was chosen as best composition for antibacterial films, as it produced the second highest tensile strength and elasticity.

### 3.6. Microstructure of Bacterial Colonies Effects

FESEM is a surface imaging method that fully capable of producing resolutions at different particle sizes, size distributions, nanomaterial shapes, and surface morphologies of synthesized particles at the micro and nanoscale. The understanding of the size, shape, surface, and aggregation state of AgNPs and SPNCCs was important to determine the films’ properties for optimizing their performance. Coatings with a high percentage of AgNPs with a smaller size were useful to strengthen the molecular bonding and minimize the bacterial colony effect on the films. The antimicrobial effects of AgNPs were known to be greatly influenced by the type and size of the AgNPs and the uncontrolled release of AgNPs or silver ions from carrier matrices [85].

According to Table 2, the length of *E. coli* and *Salmonella* and the diameter of *S. aureus* were increased after the drying process. The average length measurements of *Salmonella cholerasuis* and *Escherichia coli* from the 1 wt% and 4 wt% compositions were within 1.767 ± 0.675 to 2.90 ± 0.228 mm, respectively; meanwhile, the diameter of *Staphylococcus aureus* also showed an increasing value, with 0.556 ± 0.054 to 0.63 ± 0.0282 mm. The number of dry bacterial colonies was related to the composition of AgNPs and the storage duration. The longer the duration time, more bacterial colonies will be deactivated and the antibacterial films will slowly undergo degradation processes.

**Figure 9:**
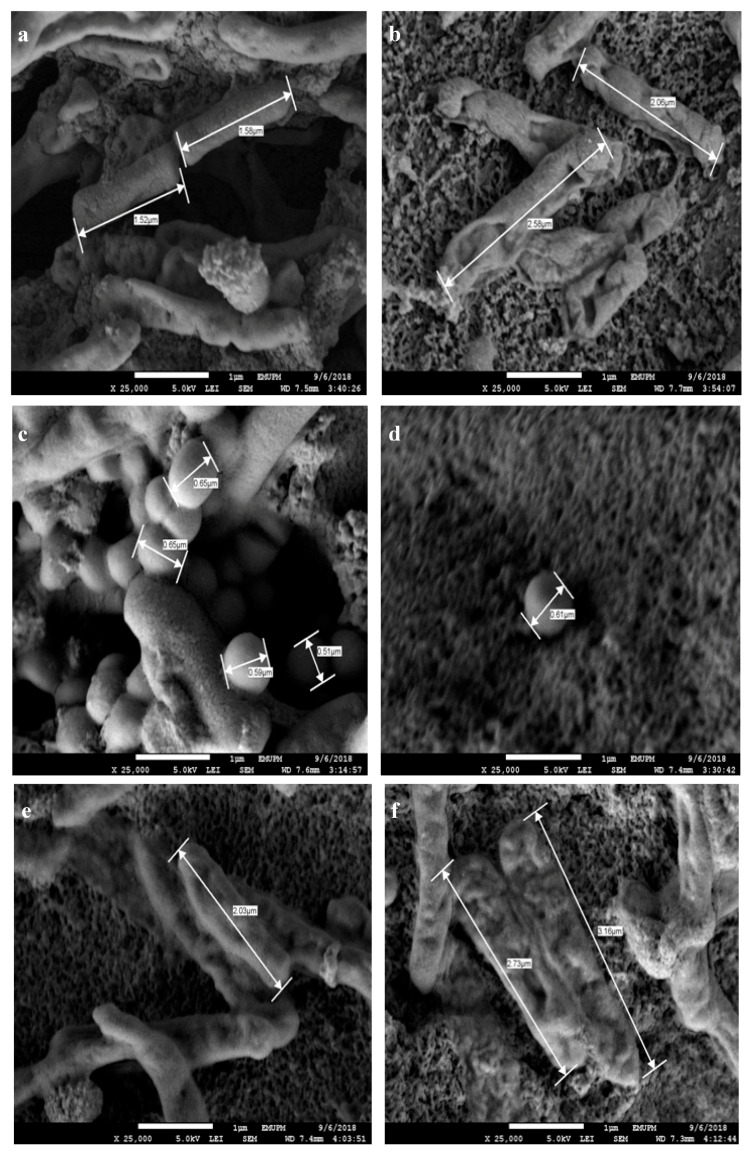
The microbe sizes: (**a**) 1 wt% *E. coli*; (**b**) 4 wt% *E. coli*; (**c**) 1 wt% *S. aureus*; (**d**) 4 wt% *S. aureus*; (**e**) 1 wt% *Salmonella*; (**f**) 4 wt% *Salmonella.*

Figure 9 shows that the length and diameter of bacteria were decreased as the cells shrank and dried. The bacterial colonies of positive (+ve) and negative (−ve) types on lower to higher compositions reduced in number and decomposed as they interacted with silver ions for several weeks. The slow dehydration during the drying process at room temperature was directly responsible for the decreased density and the reduction in bacterial colonies. The whole bacteria colony will not survive in the case of high compositions of AgNPs. The reduction in colonies and reduction in the density of bacteria at higher compositions of 3 wt% and 4 wt% showed the effectiveness of AgNPs metals by avoiding bacterial attachments. However, the 3 wt% was chosen as the best composition because the nanoparticles were the smallest and the inhibited area produced was similar than the ones achieved by the 4 wt% film.

### 3.7. AgNPs and SPNCCs Nanostructures in Antibacterial Films

The sizes of AgNPs and SPNCCs were important parameters in the determination of the antibacterial biopolymer films’ strength. Nano-sized AgNPs particles have been claimed to form strong bonds under tension and are more effective towards bacterial attack. When zooming with FESEM microscopy, most AgNPs were observed to appear brighter and smaller compared to SPNCCs. However, in the case of agglomerations, the characterization of both nanofillers resulted in a similar size, hence making them hard to identify.

Table 3 shows the nanoparticle sizes of AgNPs and SPNCCs on the core surface (matrices) and on the surfaces of the films. According to Table 3, the average combined nanoparticle sizes of AgNPs and SPNCCs on the core surface (metrics) ranged from 65.58 ± 3.43 to 97.16 ± 8.59 nm, while the average on the film surface ranged within 67.73 ± 6.39 to 94.21 ± 3.02 nm. From the table, it is observed that 3 wt% had the smallest nanoparticle size, which was due to the maximum arrangements of the molecules. Mostly, agglomeration occurred more on the surface compared to on the core surface. Agglomeration will reduce the effectiveness of the antimicrobial properties, and the strength of the film and produce inconsistent measurement values for the nanoparticle sizes.

**Figure 10:**
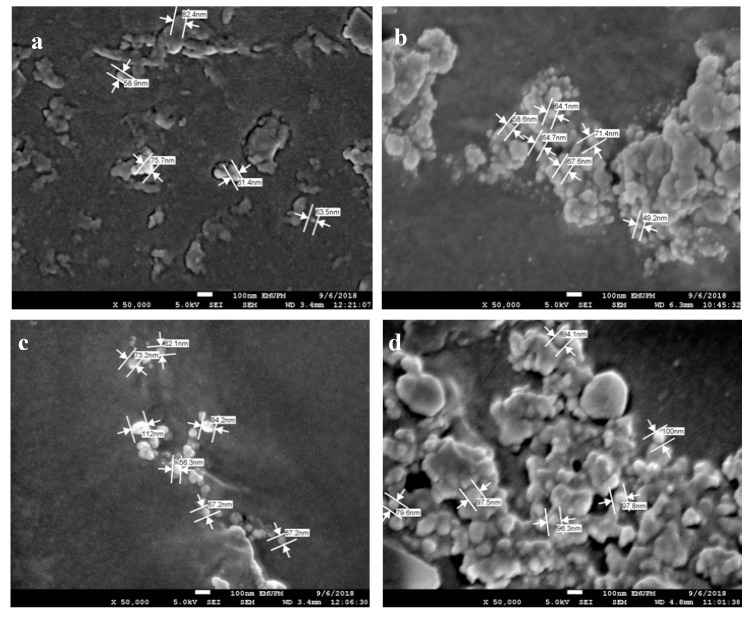

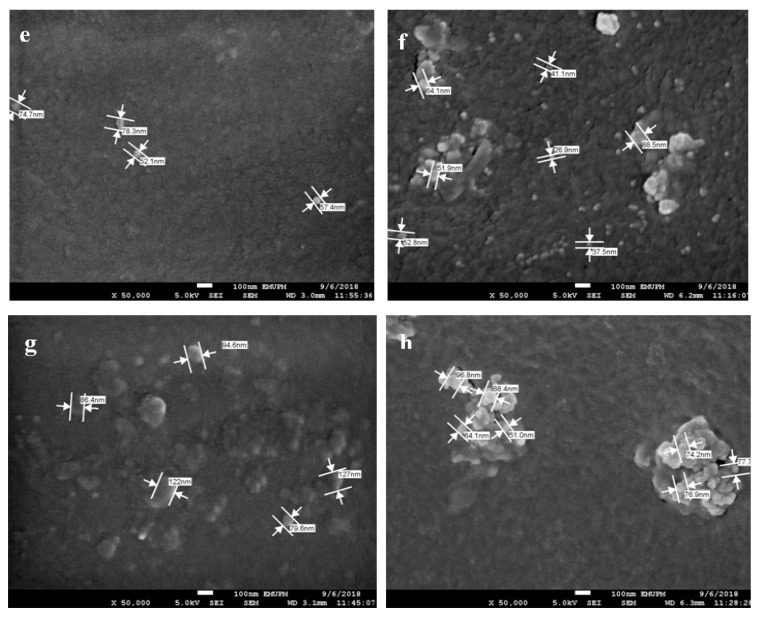
AgNPs’ and SPNCCs’ size: (**a**) core surface, 1 wt%; (**b**) surface, 1 wt%; (**c**) core surface, 2 wt%; (**d**) surface, 2 wt%; (**e**) core surface, 3 wt%; (**f**) surface, 3 wt%; (**g**) core surface, 4 wt%; (**h**) surface, 4 wt%.

Figure 10 shows the nanofiller sizes of AgNPs and SPNCCs on the core surface and on the surface of antibacterial films at 1 wt% and 4 wt%. It can be observed from the micrograph that the 4 wt% showed an increment in the nanoparticles’ sizes compared to 1 wt%, which was due to the agglomeration of AgNPs and SPNCCs, thus producing bigger molecules. The nanoparticle size of antibacterial films on the core surface was more stable in terms of the distribution, agglomerations, and measurements compared to the surface films.

In the 3 wt% compositions, the AgNPs were stabilized and the molecule arrangement distributions on the films’ matrix reached the highest molecular mixing point, thus producing the smallest nanoparticle size. Thus, 3 wt% was chosen as an ideal percentage for antibacterial films with the smallest size of nanoparticles in order to produce the higher effects on the inhibition area and a high tensile strength.

## 4. Conclusions

In conclusion, antibacterial biopolymer composite films by the addition of AgNPs in different compositions were effective in inhibiting antimicrobial attack and improving the sample films’ mechanical strength. There were some modifications on the functional groups with the addition of Ag^+^ into the formulation that caused slight changes to the intensity peaks by lowering the transmittance and shortening the frequency. The mechanical properties of the antibacterial films with AgNPs were influenced by the moisture content, weight percentage of AgNPs, and nanoparticle sizes, and agglomeration in the films matrix resulted in the ductility of the films. The diameters and lengths of the bacterial measurements were increased from 1 wt% to 4 wt% due to the effectiveness of inorganic AgNPs at higher compositions. Antibacterial biopolymer composite films with 3 wt% showed better performance in all testing, including antibacterial testing, physicochemical, mechanical properties, and microstructure images. Therefore, 3 wt% was the recommended composition for the preparation of future antibacterial films with the addition of AgNPs in food packaging applications.

## Figures and Tables

**Table 1:** Material composition of antibacterial biopolymer composite film.

Denotation of the Films	Formulation		
	Sorbitol (wt%)	Glycerol (wt%)	AgNPs (wt%)
**1**	1.5	1.5	0
**2**	1.5	1.5	1
**3**	1.5	1.5	2
**4**	1.5	1.5	3
**5**	1.5	1.5	4

**Table 2:** The length and diameter of dry bacterial colonies at 1 wt% and 4 wt%.

wt%	*Escherichia coli* (mm)	*Staphylococcus aureus* (mm)	*Salmonella cholerasuis* (mm)
1	1.804 ± 0.313 ^b^	0.556 ± 0.054 ^a^	1.93 ± 0.141 ^e^
4	1.767 ± 0.675 ^c^	0.63 ± 0.0282 ^f^	2.90 ± 0.228 ^d^

* Values with different letters in the same column are significantly different (*p* < 0.05).

**Table 3:** Nanoparticle sizes of AgNPs for different weight percentages.

Nanoparticle Size (nm)		
AgNPs Percentage (wt%)	Core Surface	Surface
**1**	66.75 ± 2.75	74.77 ± 6.56
**2**	73.78 ± 7.26	94.21 ± 3.02
**3**	65.58 ± 3.43	67.73 ± 6.39
**4**	97.16 ± 8.59	76.27 ± 3.66
